# An Overlap Syndrome of Pigment Dispersion and Pigmentary Glaucoma accompanied by Marfan Syndrome: Case Report with Literature Review

**DOI:** 10.5005/jp-journals-10008-1143

**Published:** 2013-05-09

**Authors:** Tutul Chakravarti, George Spaeth

**Affiliations:** Consultant, Eye and Glaucoma Care, Gariahat, Kolkata-700029, West Bengal, India; Research Professor, Glaucoma Services, Wills Eye Institute, Walnut, Street Philadelphia, Pennsylvania, USA

**Keywords:** Pigmentary glaucoma, Pigment dispersion syndrome, Marfan syndrome, Ectopia lentis, Intraocular pressure fluctuation, Reverse pupillary block, Exfoliation syndrome.

## Abstract

‘Overlap syndrome' describes the situation in which two or more ‘independent' conditions are present, either one of which could cause a particular finding. This current presentation reports a case with bilateral pigment dispersion syndrome (PDS), advanced pigmentary glaucoma (PG), and the Marfan syndrome, with bilateral subluxation of the lenses, and large short-term and long-term fluctuations of intraocular pressure. It is interesting to consider whether the associated advanced glaucomatous nerve damage could be a manifestation of just the PDS, just the Marfan syndrome, or rather a combination of these two overlapping independent conditions.

**How to cite this article:** Chakravarti T, George S. An Overlap Syndrome of Pigment Dispersion and Pigmentary Glaucoma accompanied by Marfan Syndrome: Case Report with Literature Review. J Current Glau Prac 2013;7(2):91-95.

## INTRODUCTION

Pigment dispersion syndrome (PDS) and pigmentary glaucoma (PG) are characterized by disruption of the iris pigment epithelium (IPE) and deposition of the dispersed pigment granules throughout the anterior segment.^1^ The classic diagnostic triad consists of corneal pigmentation (Krukenberg spindle); slit like, radial, midperipheral iris transillumination defects and dense trabecular pigmentation. The iris insertion is typically posterior, and the peripheral iris tends to have a concave configuration. The basic abnormality in this hereditary disorder remained unknown.

Marfan syndrome (chromosome 15q), an autosomal dominant disorder of connective tissue with variable expressivity, is characterized with (a) musculoskeletal features; tall, thin, scoliosis, sternal deformity, arm span > height, a narrow high arched palate; (b) cardiovascular features; dilatation of ascending aorta leading to aortic incompetence; and (c) ophthalmic features like ectopia lentis, myopia, angle anomaly. There is little discussion in the literature regarding the association between Marfan syndrome and PG. Though a case report on Marfan syndrome with bilateral PDS and an asymmetrical PG case was reported^2^ where only one eye was glaucomatous with lens subluxation, here first time a case is reported about a Marfan syndrome with bilateral lens subluxation presented with bilateral PDS and PG and advanced glaucomatous damage in very early age associated with (short-term and long-term) intraocular pressure (IOP) fluctuation.

## CASE REPORT

**Figs 1A to D:**
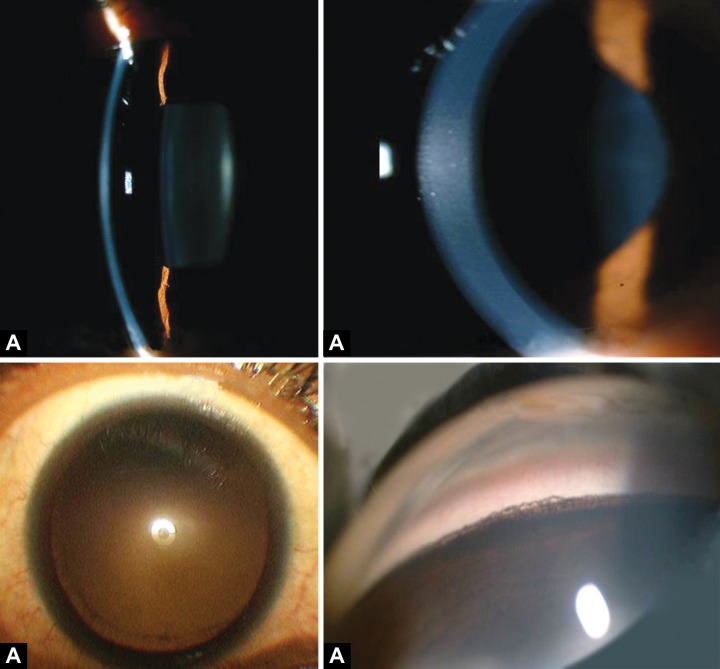
(A) Krukenberg's spindle, (B) deep AC with concave peripheral iris (C) Zentmayer line, and (D) pigmentation in angles

**Figs 2A to D:**
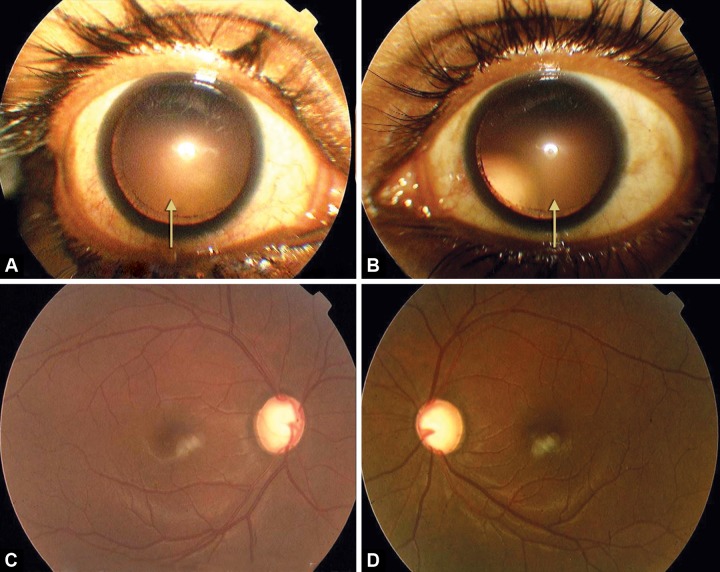
(A and B) Symmetrical temporal and upward subluxation of lens on dilation, (C and D) advanced glaucomatous cupping in both the eyes

A 22 years tall, slender Asian Indian male presented with the following findings: best corrected vision 6/6 and 6/12 with –5.00 DSph in his right and –5.50 DSph in his left eye respectively, on slit-lamp examination; bilateral Krukenberg spindle on the corneal endothelium (Fig. 1A), bilateral deep AC, (Fig. 1B; anterior chamber average centrally: 4.86 and 4.82 mm in right and left eye respectively), and patent YAG iridotomies in both eyes. His both pupils were mid dilated. Pigmented line on posterior lens capsule (Zentmayer line) at the insertion of zonule in both eyes was present (Fig. 1C). Gonioscopy with Zeiss four mirror lens revealed widely open angles, with a homogeneous, dense hyperpigmented band on the trabecular meshwork and pigment deposition on Schwalbe's line (Fig. 1D). Both eyes presented with almost symmetrical temporal and upward subluxation of lens on dilation (Figs 2A and B). On fundus examination he presented with advanced glaucomatous cupping in both the eyes (Figs 2C and D). No lattice degeneration was found in both the eyes, but areas of white without pressure (WWP) were noticed bilaterally. His visual field showed (10-2, Humphrey visual analyzer) advanced field loss. At the time of referral to our glaucoma service he was already instilling Travoprost 0.004% eye drops HS in both the eyes and his IOP was 34 and 20 mm Hg in right and left eyes, respectively. He was seen at different times of the day to record his diurnal variation and also to record his long-term and short-term IOP fluctuation. It was observed that he had a large short-term and long-term IOP fluctuation with IOP peaking mostly during the daytime.

IOP fluctuated in both eyes (with similar kind of treatment) from 10 to 34 mm Hg in right eye and 14 to 30 mm Hg in left eye. His IOP reduced to early teens on topical four topical medications.

**Fig. 3:**
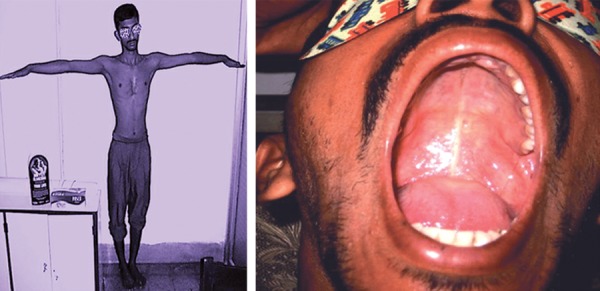
Arm span more than height and high arched palate

Cardiologist diagnosed him as a case of Marfan syndrome with the following findings: tall with some arachnodactyly; arm span more than height; (height 66 inches > arm span 69 inches), sternal deformity (sternal depression), high arched palate (Fig. 3), bilateral lenticular subluxation, mild aortic regurgitation and with mildly dilated aortic roots, partial right bundle branch block (BP 120/76 mm Hg, pulse 76/min). He was diagnosed as a case of Marfan syndrome accompanied with PG.

**Fig. 4:**
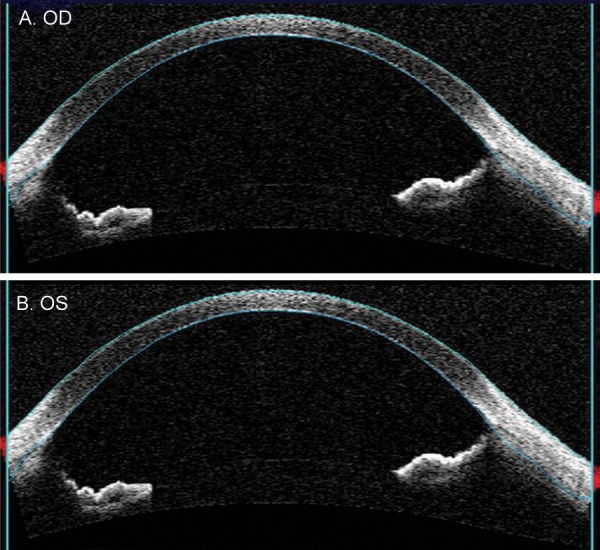
Loose zonules noted on ASOCT

Initially he was advised to instill 1% pilocarpine to reduce exercise-induced pigment dispersion and IOP elevation, but considering his loose zonules (Fig. 4) and his severe intolerance to pilocarpine, it was stopped. He was advised to follow-up after 2 months and review for further treatment option.

## DISCUSSION

PDS is a bilateral, autosomal dominant disorder and localization of a gene to chromosome 7q35-36.^3^ The mean age at the onset of PDS is probably in the mid-20s. The youngest patients reported have been 12,^4^14^5^ and 15.^6^ Although iris transillumination defect is an important diagnostic clinical feature of PDS in this patient, however, due to dark, thick iris stroma these defects were absent. Our patient had an active stage of PG according to the natural history of the disease.

There are several views regarding the pathophysiology of PG in a case of PDS which is helpful to understand the anatomical and physiological factors operating in PDS. The concept of reverse pupillary block in PG can explain the mechanism of glaucoma.^7^ The posterior bowing is eliminated by a peripheral iridotomy and by miotic therapy. It is also believed that glaucoma in PDS is related to the accumulation of pigment in the trabecular meshwork, with subsequent alteration of the trabecular beams, leading to elevated IOP and glaucomatous damage. Regarding the conversion of PDS to PG, in a long-term study, 35% patients with PDS converted to PG.^8^ The glaucoma usually develops within 15 years of the presentation of the PDS, although some follow-up averaging 27 months.^9^

Marfan syndrome, an autosomal dominant connective tissue disorder, is characterized by abnormalities of eyes, heart, aorta, skeleton, skin and lungs. Ectopia lentis is the predominant ocular abnormality and occurs in more than 60% of patients.^10^ Fibrillin plays an important role in normal capsular elasticity and, possibly, in the process of accommodation. The ectopia lentis typically appears in the fourth to fifth decade of life and is rarely complete.^11^ A study by Maumenee et al reported 422 cases of Marfan syndrome with glaucoma; all the patients belong to the age group below 40s. Though, bilateral spontaneous lens dislocation in early childhood with associated glaucoma has been reported.^12^ Glaucoma is a common ocular finding in patients with Marfan syndrome and has been reported in patients with ectopic lenses as part of the syndrome.^13^

Mechanical (lens dislocation or an anomaly of the anterior chamber angle) and vascular factors, or both, may play a role in the etiology of glaucoma in Marfan syndrome.^14^

An adrenergic hypersensitivity in patients with PDS and PG might explain large long-term and short-term IOP fluctuations and presence of mid dilated pupil in this patient.

Topical medications are recommended for the initial control of glaucoma. Surgical intervention may be inevitable in this case specially when the disease is in such an advanced stage and IOP is so unstable. Trabeculectomy is more challenging in this young myope with the greater risk of bleb failure and hypotony maculopathy.

This case describes severe glaucomatous damage in a 22-year-old male with Marfan syndrome and PDS. Prior to this case, there was only one report in the literature of an association between Marfan syndrome and PDS.^2^ Compared to the previous case report, the case described here illustrates an alternate interpretation of the association between the two syndromes. The patient reported by Doyle et al, had asymmetric PG and lens subluxation, leading the authors to conclude that the presence of asymmetric PG should prompt a search for secondary causes. In contrast, the patient characterized in this manuscript had symmetric bilateral disease and severe glaucomatous loss at a very early age, which illustrates the importance of looking for related conditions in all PDS/PG patients with advanced disease at their early age.

Neither PG nor Marfan syndrome alone can explain this unusual glaucomatous damage in this case. Two different risk factors (glaucoma due to Marfan syndrome and glaucoma due to PDS) lead to glaucomatous damage at different periods in time and by two different mechanisms. In the light of this understanding, the concept of ‘overlap syndrome' comes into one's mind.^15^ The concept of overlap syndrome was first described by Ritch (1999), who defined it as ‘the temporally sequential appearance of two or more risk factors for glaucomatous damage'. Ritch correctly emphasized that ‘the appearance of a new risk factor for glaucomatous damage can unexpectedly alter the course and prognosis of the disease'. The concept of the overlap syndrome is an attempt to create a longitudinal understanding of a patient who has glaucoma on the basis of one etiology and then develops worsening of the glaucoma by other etiology. The concept of an overlap syndrome has not previously been applied to Marfan and PG.

Glaucomatous damage in this case can be explained by the dual mechanism: (1) mechanical factors; (a) the problem of abnormal iridozonular contact of PDS and PG (exaggerated by physiological pupillary movement and accommodation), (b) the presence of zonular weakness and partial lens subluxation, common features of Marfan syndrome. (2) Perfusion abnormalities due to Marfan syndrome take into account the vascular factor.

Regarding the relationship between Marfan syndrome and PDS, Doyle et al suggested that developmental abnormalities of the iris related to Marfan syndrome caused PG.^2^ We propose that PDS and Marfan are not causally linked, but rather contribute separately to glaucomatous damage. We apply the concept of an overlap syndrome to the association between PDS and Marfan syndrome.

We have two limitations regarding using the term overlap syndrome. Timing of the two morbidities is critical to the definition of overlap syndrome. Unfortunately both the syndromes unusually appeared in this case before 20s. Ritch initially applied the concept of overlap syndrome to patients with PG who recovered from PG with age, but later developed exfoliative syndrome. Our situation is different.

Therefore we are using a modified concept of overlap syndrome.

Most patients with PDS, particularly those with mild phenotypic expression, remain undiagnosed on slit-lamp screening. Twenty-six patients were identified as having both exfoliation syndrome (XFS)/XFG and PDS/PG and in 1990, Layden and associates^16^ reported 5 such cases. Thus PDS and PG can present with XFS, Horner syndrome^17^ and even with Marfan syndrome according to this case report and case report by Doyle et al.

This case presentation demands a special attention because of the unique association of two disorders which has never been reported before. This report also advocates considering the concept of an overlap syndrome in all advanced cases of PDS/PG which can present with different combinations (like pseudoexfoliation, Horner's syndrome or with Marfan syndrome). Recognizing the potential for an overlap syndrome is very important when evaluating PDS/PG patient with advanced glaucomatous loss. For example, patients with PDS/PG patients with advanced glaucomatous loss should be evaluated regularly for Marfan, pseudoexfoliation so that the additional risk factors are noted early and properly addressed. Detection of new risk factors may allow for timely diagnosis and treatment to prevent irreversible blindness in those cases.
